# Supplementary material for: Implementation of an antibody characterization procedure and application to the major ALS/FTD disease gene C9ORF72
Source: eLife. 2019 Oct 15;8:e48363. doi: 10.7554/eLife.48363 (PMC6794092; doi:10.7554/eLife.48363)
Supplement: Supplementary file 1. [file elife-48363-supp1.docx]

| **Companies** | **Catalog number** | **RRID** | **Used in # papers** | **Citations to papers** | **Vendors proposed application(s)** | **Immunogen** | **Host/clonality** | **Wb** | **IF** | **IP** |
| --- | --- | --- | --- | --- | --- | --- | --- | --- | --- | --- |
| Abcam | ab227555 | RRID:AB_2784540 | 0 | 0 | Wb/IF-ICC/IHC | a.a.395-424 | Rabbit  polyclonal | - | - | + |
|  | ab171428 | RRID:AB_2784541 | 0 | 0 | Wb | Sequence is proprietary | Rabbit  polyclonal | - | - | - |
|  | ab121779 | RRID:AB_1845834 | 1 | 63 | IF-ICC/IHC | a.a.110-199 | Rabbit polyclonal | + | - | + |
|  | ab203627 | RRID:AB_2784542 | 0 | 0 | IHC | a.a.390-440 | Rabbit polyclonal | - | - | - |
|  | ab221137 | RRID:AB_2833081 | 0 | 0 | Wb/IF-ICC | a.a. 1-250 | Rabbit  monoclonal | +++ | - | - |
| Proteintech | PT25757 | RRID:AB_2784548 | 1 | 16 | Wb/IHC/IF | a.a. 1-221 | Rabbit polyclonal | + | - | + |
|  | PT22637 | RRID:AB_10953528 | 7 | 164 | Wb/IHC/IF | a.a. 1-169 | Rabbit polyclonal | + | - | + |
|  | PT66140 | RRID:AB_2784547 | 0 | 0 | Wb/IHC/IF | a.a. 1-222 | mouse monoclonal | - | - | - |
| GeneTex | GTX632041 | RRID:AB_2784546 | 0 | 0 | Wb/IHC/IP | Sequence is proprietary | mouse monoclonal | ++ | +++ | +++ |
|  | GTX634482 | RRID:AB_2784545 | 0 | 0 | Wb | Sequence is proprietary | mouse monoclonal | +++ | - | - |
|  | GTX119776 | RRID:AB_10617960 | 5 | 757 | Wb/IHC/IP/IF | Sequence is proprietary | Rabbit polyclonal | - | - | + |
| Sigma | HPA023873 | RRID:AB_1845834 | 10 | 1091 | IHC | a.a.110-199 | Rabbit polyclonal | + | - | + |
| Santa Cruz | sc-138763 | RRID:AB_10709750 | 15 | 3164 | Wb/IHC/IP | a.a.165-215 | Rabbit polyclonal | - | - | - |
| Cell signaling technology | CST64196 | RRID:AB_2833080 | 0 | 0 | Wb | Sequence is proprietary | Rabbit polyclonal | + | - | - |
| MRC | MRC-S478D | RRID:AB_2784544 | 0 | 0 | Wb/IP | full length | Sheep polyclonal | ++ | - | + |
|  | MRC-S479D | RRID:AB_2784543 | 0 | 0 | Wb/IP | full length | Sheep polyclonal | + | - | + |

**Laflamme et al. Supplementary File 1**
